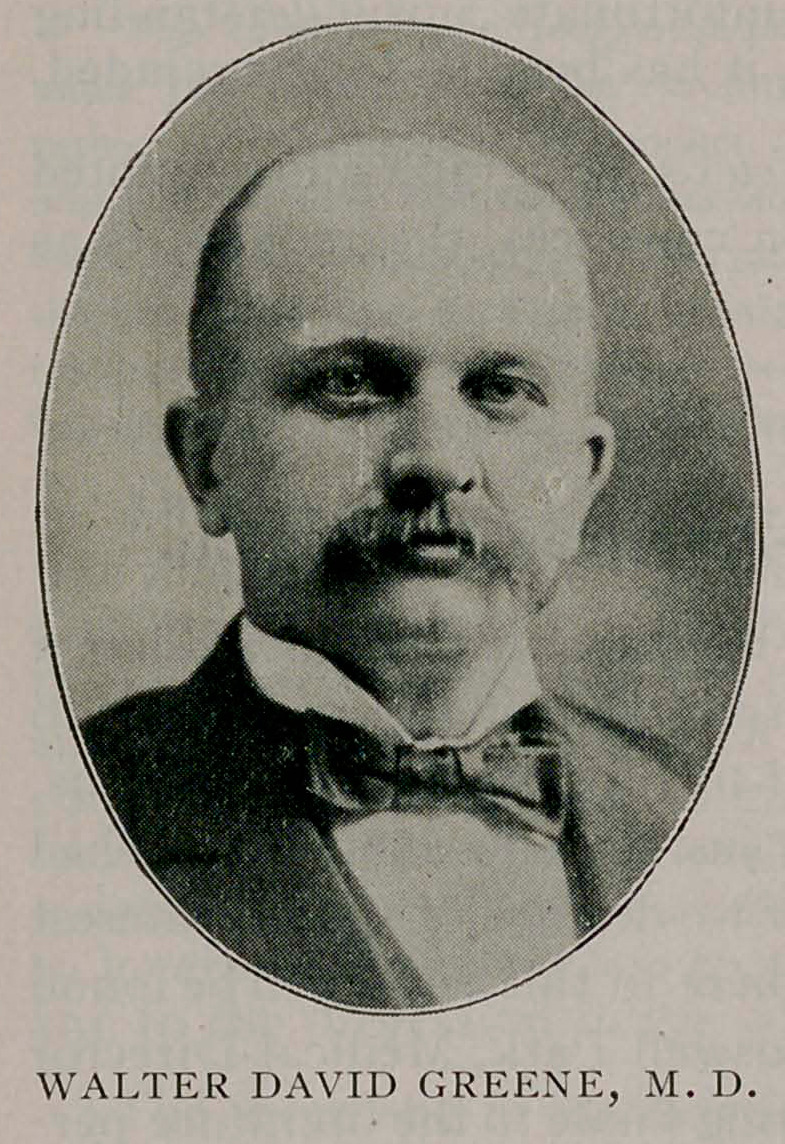# Buffalo’s New Health Commissioner

**Published:** 1902-01

**Authors:** 


### Buffalo's New Health Commissioner.

DR. WALTER DAVID GREENE, of Buffalo, was designated as Commissioner of Health by Mayor-elect Knight on or about December 1, 1901, to take office January 1, 1902, vice Dr. Ernest Wende, whose term has expired. The JOURNAL extends to Dr. Greene its congratulations and can offer him no better token of our interest in his successful administration of the office, than to wish he may prove as efficient as his predecessor and maintain the high standard that has been established by him. This does not mean that there is no room for improvement,—this is a progressive age, and improvement should be the watchword of every public officer,—and we have confidence in Dr. Greene's ability and desire to take advantage of every opportunity to increase the efficiency of his department. If he should prove a better health officer than Dr. Wende, we feel sure no one would be more pleased than the rugged and sturdy veteran who now lays off the mantle of office.

WALTER DAVID GREENE, M. D.

Dr. Greene was born in Starksboro, Vt., April 20, 1853, and received his preliminary education in the public schools in that vicinity, later teaching in those schools in intervals between his four years of intermediate education at Union Springs, N. Y., at a school now known as the Friends' School. After leaving school he obtained a position on the Duchess & Columbia Railroad, which is now a part of the Harlem division of the New

York Central Railroad, where he served as ticket agent and telegrapher.

In the fall of 1873 he came to Buffalo and commenced the study of medicine. Soon afterward he entered the medical department of the University of Buffalo, and was graduated February 23, 1876. After graduation he was appointed interne at the Rochester City Hospital, where he remained for a year and a half, and then began the practice of medicine in Mendon, N.Y. He practised there from 1877 to 1880 and then came to Buffalo. Two years later he was appointed a district physician, serving from 1882 to 1889, and then was appointed health physician. He served as chief officer of the Health Department until the end of 1891, when the department was reorganised under the new charter.

Five years later—namely, January 1, 1897, he was appointed Deputy Commissioner of Health, from which he is now promoted to Commissioner.

Dr. Greene taught hygiene and sanitary science in the Niagara University, which is now a part of the University of Buffalo. He now has a chair in that University, being clinical professor of diseases of the kidneys and bladder. He is attending surgeon at the Sisters of Charity Hospital and also at the Erie County Hospital. He is a member of the Medical Society of the County of Erie, of the Medical Union, of the Medical Society of the State of New York and the American Public Health Association.

It would thus appear that Dr. Greene has served as district physician seven years, as health physician two years, and as deputy commissioner of health five years, making fourteen years in all, and this experience must certainly prove of value to him in the office to which he has been appointed.

## Figures and Tables

**Figure f1:**